# Supplementary material for: Spatial distribution and geographical heterogeneity factors associated with poor consumption of foods rich in vitamin A among children age 6–23 months in Ethiopia: Geographical weighted regression analysis
Source: PLoS One. 2021 Jun 3;16(6):e0252639. doi: 10.1371/journal.pone.0252639 (PMC8174682; doi:10.1371/journal.pone.0252639)
Supplement: Attachment — Submitted filename: Response to Reviewers.docx [file pone.0252639.s002.docx]

**Response to Reviewers’**

***Spatial distribution and geographical heterogeneity factors associated with poor consumption of foods rich in vitamin A among children aged 6 -23 months in Ethiopia: Geographical weighted regression analysis***

Sofonyas Abebaw Tiruneh^1*^, Dawit Tefera Fentie^2^, Seblewongel Tigabu Yigizaw ^2^, Asnakew Asmamaw Abebe^2^, Kassahun Alemu Gelaye^2^.

The authors, extending our great thanks for the editors and reviewers for this manuscript as the stand of this review. The comments raised by the reviewers and editors are vital and defiantly it will improve the quality of the manuscript. We have addressed all the issues raised by the reviewers and editors point-by-point response and believed that the revised version of the manuscript is satisfactory and will meet the journal publication requirements. As well, the journal requirements amended accordingly the journal submission guideline. Please note that words and sentences highlighted by Areal font under the reviewers' question and comment were the authors' response and reaction for each issue.

**Stay Safe!!!**

The Authors.

Review Comments to the Author

Reviewer #1: It is an interesting article whose findings can help policymakers and health planners to select appropriate interventions. The methodology used can be also used to identify hot spot areas of low consumption of vitamin A and its determinants not only in Ethiopia but also in other Sub-Sahara Africa countries. However, there are some caveats and extensive editing of English language and style will be needed to facilitate the reading of the whole document.

Thank you for the comment!

Background

It is difficult to follow the different paragraph, although the background section provides sufficient information and include relevant references. The background section needs to be reorganized. It will be helpful to combine some paragraphs. For example, the first three paragraphs can be reduced to one.

Noted! Modified accordingly!

Results

Table 1: Since the study is related to children, it will be better to present children characteristics before the mothers’ results.

Noted Thank you! it was corrected accordingly.

Discussion

It will be important not to repeat most of results in the discussion section.

The consumption status of Foods rich in Vitamin A among children age 6-23 months was determined using a 24-hour recall. The results need to be analyzed with precaution, as it does not reflect any dietary habits. Thus, it needs to be highlighted in Discussion.

It will be helpful to give some examples of regional variation dietary preference, low practice to complementary feeding, or socioeconomic status.

References for this confirmation: The possible justification might be living in rural area had no access to get foods rich in vitamin A, poor knowledge about foods rich in vitamin A, and other socioeconomic factors.

Thank You! it was confirmed.

Reviewer #2: This study aimed to assess the spatial distribution and its determinants of dietary consumption of foods rich in vitamin A among children aged 6-23 months in Ethiopia. It could provide valuable information to identify areas with high vitamin A deficiency. However, there are a number of issues to be addressed.

Thank you for the comment!

Abstract

- Methods: please specify how poor vitamin A consumption was defined

Noted! Corrected accordingly!

Background

- Need references for the sentence ‘So far, different studies conducted in Ethiopia to assess dietary diversity among children including foods rich in vitamin A consumption’.

Noted corrected accordingly.

- The authors presented the importance of vitamin A in the background. However, the background regarding geospatial analysis is relatively weak. The authors should provide more details to strengthen the justification of this study. For instance, the authors indicated that there was no evidence on geospatial distribution of dietary consumption in Ethiopia. However, relevant studies were done from other countries so it would be informative to add- what is the current knowledge on geospatial distribution of dietary intake, specifically in Africa, what is the gap and how this study could contribute to the existing body of evidence. Also, if there is no study on spatial distribution of dietary consumption of foods rich in vitamin A, it would be still useful to add studies in Ethiopia targeting other nutrients or other nutrition outcomes such as stunting and wasting.

Thank you for your insight. It was modified accordingly. Even though there is no geostatistical evidence in vitamin A rich food consumption, it will be fair to discuss other nutrition status studies.

- ‘The magnitude of vitamin A deficiency (VAD) was highest in Sub-Saharan Africa (48%; 25–75) and South Asia (44%; 13–79)’: if 25-75, 13-59 mean CI or other, please specify

Thank you! it was compared with other countries or continents from the evidence of the study.

Methods and materials

- I suggest the authors revise ‘Source and study populations’ and ‘Data collection tools and procedures’ as some parts were overlapped and it is not easy to understand

It was corrected accordingly. Since the data were secondary, the method of data collection copied from the source file.

- Some important information is missing regarding the data source- for instance, how did this study handle missing data and what was the survey response rate?

The missing data by nature system missing. After extraction from the original dataset missing data was not a problem. This data set has system missing and it was done complicate case analysis. Regarding the survey response rate, so far the data were nationally representative survey and it was calculated proportionally for each region. Therefore, the survey was conducted in 16,650 residential households, 5,232 in urban areas and 11,418 in rural areas. The sample was expected to generate an estimated 16,663 completed interviews with women age 15-49, 5,514 in urban areas and 11,149 in rural areas, and 14,195 completed interviews with men age 15-59, with 4,472 in urban areas and 9,723 in rural areas.

- Outcome variable: as it is critical part in the manuscript, the authors need to provide enough details such as who responded to the question, what were the seven food items and what was the justification to define poor consumption of vitamin A

Thank you for your insight. It was corrected accordingly the comment. The operation definition was clearly stated in the manuscript. The seven food items were: Egg, Meat, Vegetables, Green leafy vegetables, Fruits, Organ meat, and Fish. If the mothers or caregivers respond for their child at least one food ‎item among the seven food items at any time in the last 24 hours preceding the interview was ‎declared good consumption of foods rich in vitamin A, if not poor consumption.

- Predictor variables: It is not clear how the Authors considered possible multicollinearity.

Multicollinearity was considered for each independent variable was checked using ArcGIS explanatory analysis. Therefore, all independent variables multicollinearity (Variance Inflation Factor <7.5) from the explanatory analysis.

- It is not clear how the authors considered complex survey design

The study design is multistage. The design was not selected by the authors because it is secondary data.

- Data management and analysis: didn’t the author also use Kuldorff’s SaTScan version 9.6 software, Arc GIS 10.7 software and MGWR (Multi-scale Geographically Weighted Regression)? If then, please indicate in addition to STATA and Excel

The data management (data cleaning) was done using STATA software and Microsoft Excel. But spatial analysis was performed using Kuldorff’s SaTScan version 9.6 software, Arc GIS 10.7 software and MGWR software.

- It is not clear what was done for model validity and uncertainty assessment. Please provide details.

The model validity assessment was assessed using AICc for best fit model selection.

- Ethical consideration: better to clearly say that ethical approval was not required for what reason.

The ethical clearance was weived form DHS data archivist after requesting a concept paper. The dataset was publically available after submitted to a concept paper.

Results

- Be consistent with presenting numbers- up to two decimals, one or? i.e. 18.45%, 61%

Noted. thank you! corrected accordingly! But for the case of "61% of mothers and 45% of husbands, 88% of children, and 45% " since it is numbering person rounding to the nearest integer is appropriate.

- Table 1: the authors need to explain how variables were classified in the ‘Methods’ section. For instance, how household wealth was classified into poor, middle and rich? Is it solely based on household income or with other assets?

The original dataset (secondary data) classify the wealth status of the household was classified as poorest, poor, middle, rich, and richest. For further analysis (Modelling) it was recategorized as Poor (Poorest and poor), middle, and rich (Rich and richest).

Please explain what Dega means.

The altitude was classified as into three categories. which as, Kolla (Tropical zone) - is below 1830 metres in elevation, Woina dega (Subtropical zone) - includes the highlands areas of 1830 - 2440 metres, and Dega (Cool zone) - is above 2440 metres in elevation. According to this classification, the Continous variable categorized as such.

And it will include in the operational definition.

Also, how ‘media exposure’ was defined?

The operation definition for media exposure declared as if the respondent has to access to listen to either radio or television said to be having media exposure.

- How about the associations with other variables such as - education, religion, occupation, child age, etc. and outcome? Please also specify if the association were not significant

Noted! These variables are not significant spatially with the outcome variable at P-value < 0.05. though it is no need to discuss.

Discussion

- It is not clear what the first paragraph is trying to say.

The first paragraph is said to be restating the prevalence of vitamin A-rich foods consumption among the study groups for internal comparison.

- The last sentence on page 19 needs more elaboration- how dietary preference, low practice to complementary feeding or socioeconomic status differ by regions and how it could explain the geographical variation of vitamin A consumption. Same goes for the last sentence on page 20.

Noted. Thank you. This sentence elaborates the situational feeding practice of the Ethiopian population. Ethiopia is a multi diversity and multiethnicity country which has different regions and nation and nationalities across each region. Besides, Ethiopia has four agrarian regions and five pastoralist region. Therefore the way to accessibility, cultural practice on their feeding will differ across this situation. Then, this might be a possible justification for the geographical variation of feeding practice among their child.

- It is not clear what ‘The possible reason might be household with poor wealth did not get minimum meal frequency to their child and poor wealth will affect adherent to the consumption of foods rich in vitamin A and dietary diversity to their child.’ means, please specify.

Noted, As we know poor wealth affects the affordability of foods for their family. Therefore, households with poor wealth status will be food insecure for their family. Then, this possible explanation will relate to this scenario. And, we put this regard as a possible explanation for poor Vitamin A rich food consumption about.

- The authors could have provided comprehensive comparison with other studies to strengthen the discussion part. For instance, what were the results of other similar studies examining the spatial distribution of food consumption or nutrition/health status? How similar or different were the results and what would be the possible reasons for that?

Noted! In such regard, there is no sufficient study on the spatial distribution of vitamin A rich food consumption. But, we try to make a comparison as maximum potential.

- The authors listed one limitation but there might be more – for instance, how food consumption was defined as poor or good? Was amount of food considered? Was there any possibility of recall bias?

Thank you, we correct accordingly.

References

- Need revision. For instance, #3 ref: (World Health Orgnaisation) is repeated

- #10 ref: year is repeated twice like ‘J Nutr Metab. 2013;2013’

Thank You! we correct accordingly.

There are some grammar and flow issues so I recommend copyediting. Below are some examples

- Venerable: do you mean vulnerable? (appeared several times in abstract, result)

- The following sentence on p9 is not clear- On the other hand, only 12 to 24% of children age 6-23 months consumed animal source foods rich in vitamin A in Ethiopia (6,12), however, eggs (11.0%) and meat (2.6%) were less frequently consumed (8).

- p20 …male frequency and Nepal which is Children from the poorest… did you mean ‘meal’?

- p21 .. The expectation of the finding of hos spot maps in line.. did you mean ‘hot’?

- p21 …This study focuses on typical consumption of foods rich in vitamin A.Tthe association between….

Noted! Thank You very much for your bird eye view review for this manuscript.

**Thank you for your constructive comment!!!**
