# Supplementary material for: Spatial distribution and geographical heterogeneity factors associated with poor consumption of foods rich in vitamin A among children age 6–23 months in Ethiopia: Geographical weighted regression analysis
Source: PLoS One. 2021 Jun 3;16(6):e0252639. doi: 10.1371/journal.pone.0252639 (PMC8174682; doi:10.1371/journal.pone.0252639)
Supplement: Attachment — Submitted filename: Response to Reviewers.docx [file pone.0252639.s003.docx]

**Response to Reviewers**

***Spatial distribution and geographical heterogeneity factors associated with poor consumption of foods rich in vitamin A among children aged 6 -23 months in Ethiopia: Geographical weighted regression analysis***

Sofonyas Abebaw Tiruneh^1*^, Dawit Tefera Fentie^2^, Seblewongel Tigabu Yigizaw ^2^, Asnakew Asmamaw Abebe^2^, Kassahun Alemu Gelaye^2^.

The authors, extending our great thanks to the editors and reviewers for this manuscript as the stand of this review. The comments raised by the reviewers and editors are vital and defiantly it will improve the quality of the manuscript. Please note that texts and sentences underneath the reviewer’s question and/or comment is the authors' response and reaction to each issue.

**Stay Safe!!!**

The Authors.

**Reviewer’s comment and question**

Reviewer #2:

Congratulations on this work. It looks greatthe and please see minor comments below.

Noted Thank you

In the introduction, the newly cited article by Pardede et al seems irrelevant (ref 15).

Thank you corrected accordingly.

• Lines 356-360: the authors mentioned that ‘This finding is not similar to a study done in Ethiopia, which found that the wealth status of the household is not statistically significant for the consumption of foods rich in vitamin A’. It would be valuable if authors elaborate more regarding the inconsistencies.

Thank you.

• Don’t need to cite figures again in discussion. No need to repeat the results – you can highlight the key findings and provide some insights.

Thank you! This might be the study period, sample size, and area difference.

• Review again the list of figures and numbering (fig 3 appeared twice in the list to and some figures were numbered as figure 1).

Thank you corrected accordingly.

• It seems that the editing wasn’t done for the final version of the manuscript because there are still some errors in the edited parts.

Corrected.

Reviewer #3: (No Response)

Thank you for your review.
